# Rabies‐Free Ghana: Scope for Improvement

**DOI:** 10.1002/hsr2.70628

**Published:** 2025-04-28

**Authors:** Sahjid Mukhida, Nikunja Kumar Das, Rajashri Patil, Sameena Khan

**Affiliations:** ^1^ Department of Microbiology Dr. D. Y. Patil Medical College, Hospital and Research Centre, Dr. D. Y. Patil Vidyapeeth Pune Maharashtra India; ^2^ Department of Microbiology DRIEMS Institute of Health Science and Hospital Kotasahi Odisha India

**Keywords:** control, deaths, dogs, Ghana, rabies

To the Editor,

We read the article “Rabies Control in Ghana: Stakeholders Interventions, challenges, and Opportunities” with deep concern [1]. The authors have correctly stated that despite multiple preventative and control tactics, this neglected tropical disease, Rabies, remains deadly and burdens the national healthcare system. More than 90% of deaths in Asia and Africa occur due to rabies [2]. Worldwide rabies death data is given on the World Health Organization (WHO) website, though there is no data for Ghana [3]. Hence, like Ghana, other African countries had a similar situation. Sadly, the current progression is inadequate to eliminate this viral zoonotic infection. Achieving Rabies zero by 30 target looks more far and mostly hard to impossible [4].

The study done by Emikpe BO mentioned the rabies data regarding cases, deaths, and vaccination from 2015 to 2018 in Ghana. These numbers show rabies status and poor compliance, though that data is almost 5 years old. Authors have to find out the data at least till the year 2020/2021 to idea of the current status and plan. Even in the Figure 1, there is no data for 2014. If there are no cases that occurred or data is not available, they should have mentioned it. From 2015 to 2019, a total of 677 suspected cases occurred as per the data given in the article [1]. As per the reports, between 2020 and 2023, a total of 793 suspected cases of rabies (including dog and other animal bites) were reported between 2020 and 2023 with 77 (9.7%) officially confirmed deaths. Last year 2023, around 331 rabies cases were registered in Ghana [5]. If we vision 2030 to make it zero need to work day and night for control as well as prevention in Ghana [1]. Also in Figure 1, when they have not got the data for livestock rabies‐positive samples, wildlife rabies‐positive samples, and bat rabies‐positive samples, they have to remove the column from the figure and just mention the number or reason behind the zero number. Just keeping the zero number for multiple parts, reduces the impact of the figures. Removing the above three parts makes the graph effective and properly readable for readers [1].

It is a very well‐known fact that canine animals including dogs are the main reservoir of the Rabies virus though another animal can be a reservoir for it. In Ghana, they are focusing only on dogs but this is not enough. They have to think about the other animal biting cases too. Just getting the data of dogs and their vaccination should be updated with the adding other animal populations who can be a reservoir for Rabies. As they have mentioned the Cat biting number for rabies infection, they have to focus on other animals too.

In Figure 2, we observe two things. One, there is total negative sample numbers are not there, and column indicators are available. If during 2015–2019, there are no negative samples, still they have to show the Zero (0) numbers in the graph. Other things, a total of 132 human cases were found positive (as per the data given in Figure 2) in 5 years between 2015 and 2019, while every year's death numbers (112) are the same. May be there were some typo mistakes or error during the graph preparation.

In the study done by Emikpe BO has mentioned the rabies vaccination status of the dogs in Ghana. Figure 3 they have to mention the % of animal vaccines administrated, as just numbers did not give an idea about the progression of vaccine trends. We have calculated and noticed that rabies vaccination in Ghana increase from 1.47% to 1.75% between 2015 and 2018 [1]. As per the government report, the number of dogs in 2023 is almost similar to the year 2019 though vaccination progress is increasing. A current report shows vaccination reaches 3% (75,000 vaccinated out of 2.5 million dogs) in Ghana [6]. We want to say this is the recent article still their data is more than 4 years old. Publically available data should be updated whenever sent for publication. Due to a lack of widespread vaccination programs against the main vectors, rabies continues to exist in our population. A shortage of resources, logistical challenges, and dog owners’ reluctance are some of the causes. The widespread usage of vaccines to reduce rabies deaths is a positive development, but this does not stop the virus from spreading across animal populations [6].

We are not blaming the author team for presenting old publically available data, though there is a still gap for improvement in the article. These unsuccessful efforts underline the importance of public health education, synchronized policies and accessible oral vaccines for dogs, animal and wildlife management, and the availability of financial budgets. We can genuinely battle the spread of rabies through such a holistic approach.

## Author Contributions

**Sahjid Mukhida:** conceptualization, writing – original draft. **Nikunja Kumar Das:** conceptualization, supervision. **Rajashri Patil:** resources, writing – review and editing. **Sameena Khan:** writing – review and editing, supervision, conceptualization.

## Ethics Statement

The authors have nothing to report.

## Conflicts of Interest

The authors declare no conflicts of interest.

## Transparency Statement

There is no use of artificial intelligence to prepared manuscript.

## Data Availability

The authors have nothing to report.
